# Difference in Hair Cortisol Concentrations between Obese and Non-Obese Children and Adolescents: A Systematic Review

**DOI:** 10.3390/children9050715

**Published:** 2022-05-12

**Authors:** Rosa Anna Kitani, Konstantina Letsou, Ioulia Kokka, Christina Kanaka-Gantenbein, Flora Bacopoulou

**Affiliations:** 1 Postgraduate Course on the Science of Stress and Health Promotion, Medical School, National and Kapodistrian University of Athens, 4 Soranou Ephessiou St., 11527 Athens, Greece; rosakitani@gmail.com (R.A.K.); dinaletsou@gmail.com (K.L.); iouliakok@med.uoa.gr (I.K.); ckanaka@med.uoa.gr (C.K.-G.); 2 Outpatient Specialty Clinic for Obsessive Compulsive Disorder and Behavioral Therapy, First Department of Psychiatry, Medical School, National and Kapodistrian University of Athens, Eginition Hospital, 11528 Athens, Greece; 3 First Department of Pediatrics, Medical School, National and Kapodistrian University of Athens, Aghia Sophia Children’s Hospital, Thivon & Papadiamantopoulou St., 11527 Athens, Greece; 4 Center for Adolescent Medicine and UNESCO Chair in Adolescent Health Care, First Department of Pediatrics, Medical School, National and Kapodistrian University of Athens, Thivon & Papadiamantopoulou St., 11527 Athens, Greece

**Keywords:** hair cortisol, stress, children, adolescents, non-obese, obese, cortisol

## Abstract

Childhood obesity has been linked to physical and psychological comorbidities that can be carried into adulthood. A bidirectional link between body weight and the stress system appears to exist, as cortisol may affect the regulation of appetite, while adiposity can affect cortisol secretion. Among the biological tissues used to evaluate cortisol concentrations, scalp hair can provide retrospective measures. The aim of this systematic review was to investigate the difference in hair cortisol concentrations between obese and non-obese minors ≤ 19 years of age. Children and adolescents with genetic, somatic or psychiatric comorbidities were excluded. The work was conducted following the PRISMA guidelines, using prespecified search terms in the Pubmed database. The initial search yielded 56 studies, while the last step of the screening procedure concluded in 9 observational studies. Among them, the results could be characterized as inconclusive. Five of them demonstrated significantly higher hair cortisol concentrations in obese children and adolescents than normal weight subjects. On the contrary, the remaining four found no statistically significant differences in hair cortisol concentrations between obese and non-obese subjects. Different methodologies applied, and confounding factors could explain the inconsistency in the findings. Further research is needed to provide more solid results.

## 1. Introduction

Childhood obesity constitutes a major health issue worldwide. According to the World Health Organization, in the last four decades its prevalence in children and adolescents aged 5 to 19 years has increased from 4% to 18% [1]. Excess weight is recognized as one of the most burdening health factors due to its association with psychological and physical comorbidities. Obese children and adolescents are more likely to suffer from depression, anxiety, lower self-esteem and behavioral disorders compared to their normal weight peers [2]. Additionally, the prevalence of physical comorbidities such as hypertension, dyslipidemia, self-reported asthma and especially non-alcoholic fatty liver disease (6.1 for overweight and 26.1 for obese children and adolescents) is higher in individuals with excess body weight than in normal weight ones [3]. Furthermore, taking into consideration that most overweight and obese minors may carry their weight status into adulthood, long term health consequences may occur [4]. Thus, the risk of developing cardiometabolic diseases, type 2 diabetes and a variety of cancer types in later life is increased in children and adolescents with an elevated Body Mass Index (BMI) [5,6].

The association between body weight and stress is well established, as reflected by the interplay of the hypothalamic-pituitary-adrenal (HPA) axis activation leading to glucocorticoids’ production, and adverse metabolic health has been found [7]. Stress hormones affect the regulation of appetite and, depending on the extent of the stress exposure, this can be expressed by either a decrease or an increase in food consumption [8]. Chronic activation of the stress system and over-secretion of its hormones increases not only appetite, but visceral fat accumulation as well, due to the alteration in the secretion of other hormones such as insulin [9]. More importantly, when adiposity is increased there is a local increase in the active glucocorticoid, cortisol, concentration; since adipocytes express higher levels of the 11-beta hydroxysteroid dehydrogenase 1 (HSD1) enzyme, that leads to the conversion of the inactive cortisol form to the active one, the cortisol [10]. Glucocorticoids (GCs) act on the adipose tissue in a dose -and time-dependent fashion, as the acute release of GCs plays a lipolytic role regarding mature adipocytes, although chronic exposure leads to hyperplasia and hypertrophy of adipocytes. The latter occurs due to GCs’ impact both on the maturation of adipose stromal cells and in storage of lipids [11]. Evidence showing higher cortisol levels in obese [12] and overweight [13] subjects when compared with normal weight participants has been established. However, contradictory findings have also been published reporting a blunted cortisol secretion in individuals with a higher BMI [14,15].

Regardless of the discrepancies between the aforementioned studies, the most used biomarkers to assess the association between weight status and cortisol level were blood serum, saliva and 24 h urine cortisol measurements. A less invasive and less time-consuming method to evaluate cortisol concentration and HPA activation in the last decades is scalp hair [16,17]. When compared to the other biological sources of cortisol concentration assessment, hair can provide long term measures of cortisol, as 1 cm of hair reflects approximately 1 month of cortisol’s secretion, without being affected by confounding factors such as its diurnal oscillations [18]. Taking into account the several advantages of this method in estimating HPA axis activity retrospectively, this review attempted to investigate the difference of hair cortisol concentration (HCC) between obese and non-obese children and adolescents.

## 2. Materials and Methods

The current systematic review investigated the difference of HCC between obese and non-obese children and adolescents. In order to conduct and report it, Preferred Reporting Items for Systematic Reviews and Meta-Analyses (PRISMA) guidelines were followed. A thorough literature search was performed in the PubMed database. The search terms used were the following: (“obesity morbid” OR “obesity” OR “obese” OR “overweight” OR “abdominal obesity”) AND (“hair cortisol” OR “long term cortisol”).

### 2.1. Eligibility Criteria

Studies were eligible for inclusion if they met exact inclusion/exclusion criteria. Inclusion criteria were as follows: subjects had to be ≤19 years of age [19]. Cortisol should be evaluated via extracted scalp hair from the posterior vertex. In addition, BMI should have been categorized according to established standards [20]. Included studies had to be published in the English language in peer-reviewed scientific journals, within the last decade. Study design did not constitute a criterium of eligibility. With respect to the exclusion criteria, studies of both minors and adults, without stratifying data by age group were excluded. Children and adolescents with genetic, somatic or psychiatric comorbidities were also excluded. Other systematic reviews or meta-analyses were excluded, as well as research protocols without reports of their results.

### 2.2. Search Strategy

Titles, keywords and abstracts of articles published within the last decade were initially screened. Following this, a full-text reading was conducted for those papers meeting the eligibility criteria. The characteristics of the included studies were then tabulated on a Microsoft Excel worksheet, in which the following information was reported: title/authors; year of publication; characteristics of participants (sample size, mean age, BMI status); method of hair cortisol assessment and length of hair samples; and main outcomes regarding this review’s research question.

## 3. Results

### 3.1. Study Selection

**Figure 1:**
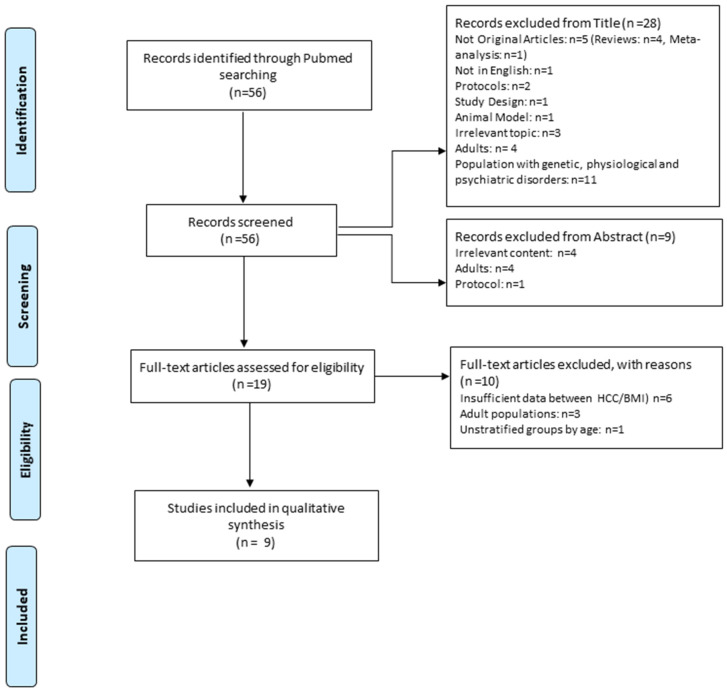
Flow diagram of the study selection.

The initial search yielded 56 results. Most articles were rejected by the title since they contained a sample of genetic, organic or psychiatric illnesses [12], while five were not original studies, two were protocols, one was the description of the researchers’ study design, one was performed in animal models, three were not relevant to what was being reviewed in the present systematic study, four studies included adults and one was published in a language other than English. Then, based on the summary, a total of nine studies were rejected, of which four were related to an irrelevant topic, three concerned the adult population and one concerned the publication of a study protocol. Finally, based on the full text, 10 studies were rejected. The complete screening procedure of the study selection is presented in Figure 1. These studies compared the concentration of hair cortisol between children and adolescents with or without obesity.

### 3.2. Basic Characteristics of the Included Studies

The nine studies included in this review recruited 5.794 subjects in total, of whom 2.796 were of normal weight, and 799 overweight or obese. One of the studies (n = 2.037) did not clarify the subgroups’ sample sizes with respect to weight. Among them, the mean age was 7.93 years while the mean female participation was 58.4% for eight of the studies, with one of them including solely female subjects. Five of the studies were conducted in Europe, while the remaining four were divided in the USA and Australia. Regarding the study design, all of them were observational, with seven out of nine being cross-sectional studies. Eight of them used the International Obesity Task Force (IOTF) criteria for establishing the cut-off point for obesity, while one applied the CDC criteria. Each study’s characteristics are presented in Table 1.

### 3.3. Hair Cortisol Concentrations

In the study by Veldhorst et al. where children of 8 to 12 years of age were included, the group of obese subjects demonstrated higher hair cortisol concentrations (25 [17.32] pg/mg) than normal weight children (17 [13.21] pg/mg), and this difference was statistically significant (*p* < 0.05). Hair cortisol concentrations were assessed by Enzyme-linked immunosorbent assay (ELISA) and the kit used was the ELISA saliva cortisol kit (DRG GmbH) [22]. The study of Papafotiou et al. concluded similar results; a statistically significant difference (*p* < 0.0001) in hair cortisol concentrations between obese (4.1 ± 5 pg/mg) and normal weight subjects (1.2 ± 0.6 pg/mg) was found [23]. However, their sample consisted solely of prepubertal females, to reduce the effect of adolescence and sex steroids on cortisol’s metabolism. Cortisol concentrations were measured by liquid chromatography-mass spectrometry (LC-MS/MS) using the Xevo TQ-S system [23]. In the study of Noppe et al. a sample of 2953 six-year-old children from the Generation R study were included and HCC exhibited statistically significant association among the groups of obese and overweight individuals (OR: 9.4 [3.3–26.9], OR: 1.39 [1.0–2.0] respectively) [24]. Part of this sample (n = 2037) was recruited prospectively at the age of 10 from the study of Vehmeijer et al. Children who exhibited elevated HCC at the age of 6 had a higher risk of obesity and overweight in the time of the study (95% CI: 0.10 [0.04, 0.16], *p* < 0.017) [25]. Both studies conducted the hair cortisol analysis with LC-MS/MS using the Xevo TQ-S system [24,25]. Similarly, Distel et al. concluded that children with a higher BMI had higher HCC (r = 0.33, *p* = 0.02). The measurements of cortisol concentrations in this study were conducted with ELISA, using the EIA kit [26].

In the remaining four studies, no statistically significant difference between the HCCs of normal, overweight and obese children and adolescents was found. Participants of the Genitsaridi et al. study were aged between 4 and 18 years. The largest percentage of the sample belonged to the groups of overweight (31.3%) and obese (46.7%), and the group with the highest concentration of hair cortisol was that of the normal weight. The method used to calculate the HCC was with the automatic electrochemiluminescence analyzer Cobas e411 from Roche Diagnostics GmbH (measurement “Cortisol II”). The results of the study did not show a statistically significant difference in HCC among the different BMI groups (*p* = 0.733) [27]. The results of the study of Olstad et al. showed that BMI was not statistically significantly associated with hair cortisol concentration (*p* = 0.739). The BMI groups were divided into normal, overweight and obese children/adolescents. The obese group made up 10% of the sample, the overweight 20% and the normal weight 70%. The method used to calculate HCC was Enzyme-linked immunosorbent assay-ELISA (Salivary ELISA Cortisol kit, Alpco Diagnostics, Windham, NH, USA) [28]. In the study of Bryson et al. the question of whether the adversity risk could lead to higher HCC (physical stress), and therefore to health outcomes (including elevated BMI), was examined. However, no association among the BMI z score of overweight and obese participants with HCC was found (β = 0.76, 95% CI: 0.51, 1.12, *p* = 0.16). Hair cortisol concentration was measured with the use of ELISA (Salimetrics; Carlsbad, CA, USA) [29]. The last study included in this review was that of Ling et al. Researchers investigated the relationship of HCC of preschoolers of low-income mothers and BMI z score. Even though a small positive relationship between the variables was found (β = 0.01, 95% CI: 0.002, 0.02), this was not statistically significant (p = 0.112). Similarly to the majority of the studies included in this review, the chosen method for cortisol evaluation was the ELISA (Arbor Assays DetectX) [30].

## 4. Discussion

This systematic review intended to reveal possible differences in HCC between obese and non-obese children and adolescents. The included studies were observational and more specifically of cross-sectional, prospective cohort and longitudinal design, which investigated HCC among non-obese and obese minor subjects. Findings could be characterized as contradictory and inconclusive, as five out of nine studies reported significant differences between the groups in comparison.

One of the possible reasons for the reported inconsistency could be the different research design in investigating this relation. Furthermore, the heterogeneity of specific demographic characteristics, including age and sex, could be an additional plausible explanation. According to the existing literature, the adult stress response varies between sexes, due to the bidirectional relationship of gonadal and HPA axis hormones [31,32]. These differences in HPA axis activity are equally present during childhood, and, according to recent meta-analytic findings, are depended on the pubertal stage. Prepubertal boys under the age of 8 years (based on Tanner staging) had higher levels of serum and saliva cortisol when compared to peer females and vice versa; females of 8–18 years had higher levels of the same biomarker, while 24 h urine cortisol seemed to be higher in males at all ages [21]. In addition, HPA axis reactivity seemed to differ between sexes, as the diurnal secretion rhythm of cortisol demonstrated a greater variance in females when compared to males [33]. Papafotiou et al. recruited only female subjects of the prepubertal stage in order to eliminate confounding factors related to sex and age, and concluded that obese females were demonstrating elevated HCC in comparison to normal weight females [23]. Similarly, in the study of Noppe et al. which included the largest sample of all studies (n = 2953), 6-year-old children of both sexes in an almost equivalent analogy (48.1% boys, 51.2 % girls) were recruited, leading to a statistically significant association among obese and overweight participants and HCC [24]. These results seem to be consistent over time for those children with higher BMI; Vehmeijer et al. conducted a prospective cohort study by recruiting the same participants at the later age of 10 years, and concluded that those exhibited greater HCC [25].

Veldhost et al. also found higher HCC in the obese than the normal weight group, and even though subjects were of both sexes, 75% of the sample were girls [22]. Moreover, most of the obese children in the study of Veldhorst et al. [19] were of a different ethnicity, a factor that was not controlled in the analysis. Correspondingly, in the study of Ling et al. most of the sample of low-income preschoolers were of Black race (60%) and 24% were Hispanic. However, the sample consisted at most of normal weight children (62.9%), than overweight (14.3%) and obese (20%), and even thought there was a positive association between HCC and BMI z score, it was not statistically significant [30]. In another study investigating similar outcomes, it was shown than HCC in 1-year-old Black children was higher than in 1-year-old white children [34]. On the other hand, Distel et al. examined HCC specifically in low-income Mexican American children, and those of higher BMI had elevated HCC. However, the sample consisted of more abnormal weight children (25.0% overweight, 29.3% obese) than normal weight (44.6%) and also most of the subjects were females (61%) [26].

Some of the studies included participants facing social adversities such as food insecurity, which could probably act as a stressor, and thus result in higher HCC which may affect fat accumulation [26,28,29,30]. In the majority of these studies, HCC did not exhibit an association with BMI; yet, in all of them, the study groups consisted mostly of normal weight participants [28,29,30]. Only the study of Distel et al. found that not solely migrant children with excess weight have greater HCC, and that food insecurity was only related with elevated BMI when HCC appeared higher. In addition, even though their sample included solely 52 children, the subgroup of the abnormal weight participants had the largest [26]. In accordance with a recent meta-analysis, migration was linked to a higher prevalence of overweight and obesity in children. However, studies examining food insecurity as a contributing factor to elevated BMI exhibited contradicting results [35]. Although the evidence of the association between social adversity and children’s HCC is inconsistent, there is evidence that a positive association does exist [36]. When HCC is analyzed, researchers make the assumption that the hair growth rate is approximately 1 cm per month. There seems to be a difference in hair growth parameters between African and Caucasian hair; African hair density is lower, and hair growth rate is slower compared to Caucasian hair [37]. Taking this difference into consideration, including subjects of different ethnicities may have affected the results [38].

Another factor that may influence hair cortisol concentrations is the child’s waist circumference and fat mass, as shown in a recent meta-analysis [39]. A systematic review found that fat distribution will most likely lead to different cortisol profiles even if the BMI is considered as clinically within the normal range. Subjects with a low BMI but high levels of abdominal fat will possibly exhibit an HPA axis dysregulation, as observed in the review’s findings. More specifically, the cortisol awakening response, dexamethasone suppression test and cortisol reactivity were at times linked to the waist-to-hip ratio and not to BMI [40]. In this review, not all studies examined fat distribution [26,28,29,30]. However, in order to calculate fat mass, Noppe et al. used dual-energy X-ray absorptiometry (DXA) [24], and then prospectively in the study of Vehmeijer et al. not only was fat mass measured by the same means, but also organ and visceral fat via magnetic resonance imaging (MRI) [25], which are way more sensitive tools to evaluate fat accumulation [41]. In contrast to all studies included in this review, Olstad et al. did not measure the waist-to-hip ratio. Moreover, there were other factors such as age range and the small number of obese (n = 3) and overweight (n = 6) minors within a rather small sample of 30 subjects, that could have led to the absence of significant results in HCC among groups [28]. Studies reporting significant differences in HCC between groups of different BMIs revealed similar results when comparing waist-to-hip ratio, fat mass and liver fat fraction but not with visceral and organ indices.

The only study that found no statistically significant difference between HCC of normal weight, overweight and obese children and adolescents, and the same result occurred regarding waist-to-hip ratio and HCC among groups was that of Genitsaridi et al. [27]. Cortisol levels have been found to exhibit diurnal fluctuations, which are normally higher in the morning and lower during the evening time in accordance with the well-known circadian rhythm of cortisol secretion [42]. Excess weight disrupts this pattern of daily oscillations, as previous research has reported a reverse circadian activity in obese compared to normal weight children and adolescents, with higher salivary cortisol levels in the evening and lower in the morning [43]. On the contrary, other findings have reported lower HCC for non-normal weight minor groups at all times [44].

Additionally, a confounding parameter that should be taken into account is that researchers performed different methods when measuring hair cortisol concentrations [45]. Even when the method followed was the same, e.g., the immunoassay method of ELISA, the kits used to analyze the sample were different. Furthermore, different protocols were executed during the analyses, even with respect to hair sample selection. A recent study, comparing two LC-MS/MS methods with four immunoassay methods when testing HCC of hair samples from the same person in different laboratories, found the correlations among methods were high. However, due to steroid cross-reactivity such as cortisone, that differs among four immunoassays manufacturers, the absolute values of HCC varied and in comparison to LC-MS/MS were higher [46]. As 11β-HSD1 isoform is expressed in hair follicles and can convert cortisone in cortisol, the immunoassay’s cross-reactivity with cortisone can affect HCC by increasing it. In addition, differences in the extraction process may result in different HCC, i.e., milled hair in comparison to finely cut hair with repeated incubation in methanol and acetone exhibit lower HCC [47]. Taking into consideration that LC-MS/MS is not affected by cross-reactivity and that it is a more sensitive method than immunoassays in steroids’ assessment [48], studies that applied this method were considered more reliable in HCC measurements, and all exhibited an association among elevated HCC and BMI [23,24,25].

In one of the included studies, researchers provided the parents with hair extraction kits, and thereby the protocol could not have been ensured. The collection of hair is preferred to be as close to the scalp as possible, and this cannot be ensured when the procedure is not performed by qualified researchers [28]. These methodological differences in measuring HCC make the comparability of study results questionable.

## 5. Conclusions

The results of this systematic review revealed a possible association among higher HCC and elevated BMI; however, the results are inconclusive. Five out of nine studies found a statistically significant association in HCC between obese and non-obese subjects, whereas the other four found no difference. These contradictory results reflect the different study design and methodology as well as comparability of study results. HCC can be influenced by several factors, most of which should be taken into consideration. It is known that exposure to social stress, race/ethnicity, pubertal stage, gender, mental health (of the child and the parents) and fat distribution are some of the parameters that may affect the study results. Future research focusing on eliminating these confounders and also using more sensitive methods of cortisol analysis is needed to conclude more sufficient and valid results about long term cortisol concentrations among children and adolescents of different BMI statuses. More prospective longitudinal studies with large samples of participants in all BMI statuses could provide researchers with more sustainable evidence about HCC alterations among non-obese and obese individuals.

## Figures and Tables

**Table 1:** Basic characteristics of the studies included in this review.

First Author, Publication Year, Country	Study Design	Sample Size (n)	Mean Age (SD), Sex Distribution	Sample Distribution Based on BMI, Mean BMI & SD (Where Applicable)	Obisity Criteria	Method of Assay	Main Results
Genitsaridi et al., 2019, Greece	cross-sectional	300	10.49 (±0.15) Female n = 224 Male n = 76	Normal weight n = 66, 20.40 ± 0.32 Overweight n = 94, 23.49 ± 0.20 Obese n = 140, 29.60 ± 0.42	IOTF	ECLIA	HCC obese = 8.74 ± 0.43 pg/mg hair. HCC overweight = 8.88 ± 0.52 pg/mg hair. HCC normal weight = 9.33 ± 0.72 pg/mg hair (*p* = 0.733). HCC did not exhibit significant differences among children with different BMI status.
Veldhorst et al., 2013, The Netherlands	cross-sectional	40	Obese: (10.8 ± 1.3) Normal weight: 10.8 (±1.2) Female n = 30 Male n = 10	Normal weight n = 20, 16.4 ± 1.6 Obese n = 20, 29.6 ± 4.9	IOTF	ELISA	HCC obese = 25 [17,21] pg/mg. HCC normal weight = 17 [13,22] pg/mg hair (*p* < 0.05). (Median [interquartile rage]). Children with obesity had higher HCC than normal weight children (*p* < 0.05).
Olstad et al., 2016, Australia	cross-sectional	30	14.3 (±3.9) Female n = 13 Male n = 17	Normal weight n = 21, N/A Overweight n = 6, N/A Obese n = 3, N/A	IOTF	ELISA	HCC averaged = 96.6 ng/g (SD 49.6 ng/g). Association between HCC and zBMI: β = 0.15 95% CI (−0.76, 1.06) (*p* = 0.739). No significant association was found between zBMI and HCC (*p* = 0.739).
Papafotiou et al., 2017, Greece	cross-sectional	50	7.6 (±1.3) Female n = 50 n = 25 non-obese n = 25 obese	Normal weight n = 25, 17.2 ± 1.8 Obese n = 25, 24.6 ± 3.4	IOTF	LC-MS/MS	HCC obese = 4.1 ± 5 pg/mg hair. HCC normal weight = 1.2 ± 0.6 pg/mg hair (*p* < 0.0001). Prepubertal girls with obesity had increased HCC in comparison to normal weight prepubertal girls.
Noppe et al., 2016, The Netherlands	cross-sectional	2953	6.2 (±0.6) Female n = 1532 Male n = 1421	Non-obese n = 2825 Obese n = 128 16.2 ± 1.9 (mean, sd BMI for the entire sample)	IOTF	LC-MS/MS	HCC obese = (OR’s): 9.4 (3.3–26.9) for highest cortisol quintile. HCC overweight = (OR’s: 1.39 (1.0–2.0) for highest cortisol quintile. The association between BMI and HCC was statistically significant (*p* < 0.0001).
Bryson et al., 2019, Australia	cross-sectional	297	3.1 (±0.1) Female n = 180 Male n = 117	Underweight n = 14, N/A Normal weight = 198, N/A Overweight n = 49, N/A Obese n = 14, N/A	IOTF	ELISA	HCC mean = 8.5 (7.8) pg/mg. (SD) [range] = [1.1–45.5]. HCC obese/overweight = (β = 0.76, 95% CI, 0.51–1.12). The association among BMI z score of overweight/obese group was not statistically significant *p* = 0.16.
Vehmeijer et al., 2021, The Netherlands	prospective cohort	2037	5.9 (5.7, 8.0) Female n = 1072 Male n = 970	Underweight Normal weight Overweight Obese	IOTF	LC-MS/MS	HCC median (95% range) = 7.30 (2.64, 29.03) pg/mg. HCC and association with BMI at age 10 = (β = 0.10, 95% CI, 0.04, 0.16) SDS. The association between HCC and BMI was statistically significant *p* < 0.017.
Distel et al., 2019, USA	longitudinal	52	8.36 (SD: n/a) Female n = 61% Male n = 39%	Νormal weight = 44.6%, N/A Overweight = 25%, N/A Obese = 29.3% N/A	NA	ELISA	HCC = 0.53–369.60 pg/mg (SD= 63.44). HCC levels were higher among groups with higher BMI (r = 0.33, *p* = 0.02).
Ling et al., 2020, USA	cross-sectional	35	4.69 (±0.78) Female n = 17 Male n = 18	Underweight n = 1 Normal weight n = 22 Overweight n = 5 Obese n = 7, (mean, sd BMI for the entire sample) 16.62 ± 1.65	CDC	ELISA	HCC range = 0.5–157.2. HCC was positively associated with BMI z score of preschoolers (β = 0.01, CI 95% −0.002, 0.02), but not statistically significant *p* = 0.112.

**Abbreviations**: HCC = Hair Cortisol Concentration, SD = Standard Deviation, IOTF = International Obesity Task Force, ECLIA = Electrochemiluminescence Immunoassay, ELISA = Enzymed-Linked Immunosorbent Assay, LC-MS/MS = Liquid Chromatography Tandem Mass Spectrometry pg = Picogram, mg = Milligram, ng = Nanogram, OR = Odds Ratio, CDC = Centre for Disease Control and Prevention, N/A = Not Applicable.

## Data Availability

Not applicable.
